# Development of a single base extension method to resolve Y chromosome haplogroups in sub-Saharan African populations

**DOI:** 10.1186/2041-2223-1-6

**Published:** 2010-09-01

**Authors:** Thijessen Naidoo, Carina M Schlebusch, Heeran Makkan, Pareen Patel, Rajeshree Mahabeer, Johannes C Erasmus, Himla Soodyall

**Affiliations:** 1 Human Genomic Diversity and Disease Research Unit, Division of Human Genetics, School of Pathology, University of the Witwatersrand and the National Health Laboratory Services, Johannesburg, South Africa

## Abstract

**Background:**

The ability of the Y chromosome to retain a record of its evolution has seen it become an essential tool of molecular anthropology. In the last few years, however, it has also found use in forensic genetics, providing information on the geographic origin of individuals. This has been aided by the development of efficient screening methods and an increased knowledge of geographic distribution. In this study, we describe the development of single base extension assays used to resolve 61 Y chromosome haplogroups, mainly within haplogroups A, B and E, found in Africa.

**Results:**

Seven multiplex assays, which incorporated 60 Y chromosome markers, were developed. These resolved Y chromosomes to 61 terminal branches of the major African haplogroups A, B and E, while also including a few Eurasian haplogroups found occasionally in African males. Following its validation, the assays were used to screen 683 individuals from Southern Africa, including south eastern Bantu speakers (BAN), Khoe-San (KS) and South African Whites (SAW). Of the 61 haplogroups that the assays collectively resolved, 26 were found in the 683 samples. While haplogroup sharing was common between the BAN and KS, the frequencies of these haplogroups varied appreciably. Both groups showed low levels of assimilation of Eurasian haplogroups and only two individuals in the SAW clearly had Y chromosomes of African ancestry.

**Conclusions:**

The use of these single base extension assays in screening increased haplogroup resolution and sampling throughput, while saving time and DNA. Their use, together with the screening of short tandem repeat markers would considerably improve resolution, thus refining the geographic ancestry of individuals.

## Background

The Y chromosome has demonstrated its utility, for a number of years, in shedding light on human history and identifying population affinities. Given that human genome variation evolves over time due to several factors - among them mutation, genetic drift, migration and selection - the genome has retained some of the record of these historical and evolutionary events. This record is more easily read from the Y chromosome due to the lack of recombination along most of its length and a strict paternal mode of transmission. Consequently, the Y chromosome has become a marker of the male contribution to the shaping of human populations and their histories.

A standard nomenclature established by the Y Chromosome Consortium [1] resolved the global pattern of Y chromosome variation into 18 major haplogroups that were classified using capital letters A through to R. This has recently been revised by Karafet *et al. *[2] to a Y chromosome haplogroup phylogeny that contains 311 branches delineated by approximately 600 markers (primarily bi-allelic) and includes an additional two haplogroups (S and T), increasing the major haplogroup number to 20. The frequency and distribution of these haplogroups shows good concordance with the geographic distribution of populations. This, together with high levels of population differentiation, [3] have added value to the Y chromosome as a tool for reconstructing the history and migrations of humans over time.

While Y chromosome short tandem repeats (STRs) are now used routinely in forensic analysis [4], the use of bi-allelic markers - mainly single nucleotide polymorphisms (SNPs) - which designate Y chromosome haplogroups, is advancing steadily due to their ability to provide information on the geographic origin of individuals. Their use, however, is hindered by the paucity of simple screening methods and insufficient knowledge of their global distribution. However, this has improved in recent years [5]

A number of assays for the rapid screening of Y chromosome haplogroups have been developed [6-9]. These were targeted primarily at resolving the major haplogroups found in European populations. While these studies have included in their assays a few SNPs to resolve the major Y chromosome haplogroups commonly found in sub-Saharan Africa, they do not contain the markers needed to resolve the majority of terminal branches of the Y chromosome phylogeny that exist among African populations.

In the present study, we report on the development of single base extension (SBE) assays used to refine the resolution of Y chromosome haplogroups commonly found in Africa, having also incorporated a few SNPs to delineate the common non-African Y chromosomes following a hierarchical screening process. SBE, due to its convenience and relative affordability, is now used in many genetic and forensic applications. Following the validation of the assays, we applied these methods in order to resolve the Y chromosome haplogroups in 683 male subjects, primarily from southern Africa. Haplogroup frequencies for the populations analysed were then calculated.

## Results and discussion

### SNP selection and screening strategy

**Figure 1:**
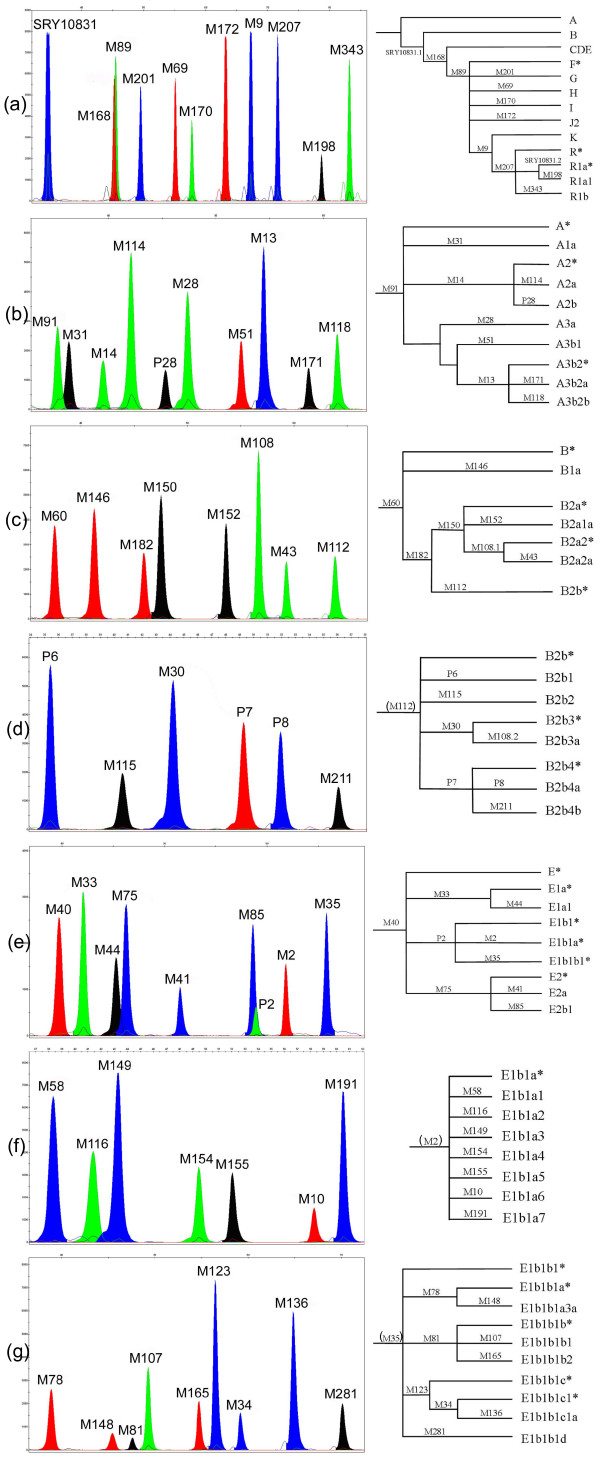
**Electropherogram and phylogeny of (a) YSNP1, (b) Hg-A, (c) Hg-B, (d) Hg-B2b, (e) Hg-E, (f) Hg-E1b1a, and (g) Hg-E1b1b1**. The markers shown in parentheses are there to indicate the hierarchical nature of the phylogenies, and are not a part of the panels they are shown with.

Seven multiplex SBE assays, which incorporated 60 Y chromosome markers described in the YCC Phylogeny 2003 [10], were developed which resolved 61 Y chromosome haplogroups. The first multiplex, YSNP1, consisted of the markers SRY10831, M168, M89, M201, M69, M170, M172, M9, M207, M198 and M343 (Figure 1a). YSNP1 resolved Y chromosomes into either the African haplogroups (A, B or E) or Eurasian haplogroups found occasionally in African males [unpublished restriction fragment length polymorphism (RFLP) data]. Note: the marker, SRY10831, initially resolves haplogroup BR, while its reversion is used to define haplogroup R1a.

Any sample found to harbour the ancestral state at all markers within YSNP1 was screened using the multiplex assay, Hg-A. This multiplex consisted of the markers, M91, M31, M14, M114, P28, M28, M51, M13, M171 and M118 (Figure 1b) and was used to resolve the sub-clades of haplogroup A. Samples found to be derived at SRY10831, but ancestral at all other markers within YSNP1, were screened using the multiplex assay, Hg-B. This multiplex consisted of M60, M146, M182, M150, M152, M108, M43 and M112 (Figure 1c) and resolved the sub-clades of haplogroup B. Those samples with the derived allele at M112 were screened further, using the multiplex assay, Hg-B2b, which contained the markers P6, M115, M30, P7, P8 and M211 (Figure 1d), providing resolution of haplogroup B2b samples to the terminal branches of the phylogeny. While M108 recurs in haplogroup B2b resolving haplogroup B2b3a, its presence in the Hg-B multiplex assay should be sufficient to resolve both its occurrences in haplogroup B, thus negating the need to include it in the Hg-B2b assay.

Those samples found to be derived at SRY10831 and M168, while remaining ancestral at all other markers within YSNP1, could be assigned to haplogroups C, D or E. These samples were then screened using the Hg-E multiplex assay, which consisted of M40, M33, M44, M75, M41, M85, P2, M2 and M35 (Figure 1e). Samples found to be derived for M2 or M35 would fall into haplogroups E1b1a or E1b1b1, respectively. E1b1a Y chromosomes were further resolved using the assay, Hg-E1b1a; a multiplex comprised of the markers M58, M116, M149, M154, M155, M10 and M191 (Figure 1f). Those Y chromosomes assigned to haplogroup E1b1b1 were screened further using the multiplex assay, Hg-E1b1b1, which consisted of the markers M78, M148, M81, M107, M165, M123, M34, M136 and M281 (Figure 1g). When a sample was found to be ancestral for the M40 polymorphism, it was screened for the mutations that defined haplogroups C (M130) and D (M174) separately. This hierarchical screening approach facilitated the resolution of the relevant haplogroup in an individual after one, two, or at most, three reactions, depending on the haplogroup present.

### Polymerase chain reaction (PCR) optimization

While PCR primer concentrations were initially 0.02 μM - 0.04 μM, these were increased or decreased incrementally in order to obtain a relatively equal amplification of amplicons in the multiplex PCR (see Table 1). The marker P28, in the Hg-A assay, initially experienced low amplification after multiplexing. This was rectified by increasing the final concentration of the P28 PCR primers to 0.2 μM, and decreasing the buffer concentration to 0.8×. The annealing temperature was also optimized in order to ensure maximum product yield and to minimize the formation of spurious products. A spurious amplification product was found to occur in the Hg-E1b1b1 assay which was eliminated by increasing the annealing temperature to 61°C.

**Table 1:** Polymerase chain reaction (PCR) primer sequences, amplicon lengths and final PCR primer concentrations used in the study.

SNP	Primer (5' - 3')		Fragment size (mers)	Concentration (μM)	Assay
				
	Forward	Reverse			
M170	CTAGTATGCTTCACACAAATGCG	GACCACACAAAAACAGGTCCTC	390	0.08	YSNP1
M207	AAGGGCAAGCAAAATAGCAATAC	TGTTCGCTGCTACGAATCTTT	363	0.08	
M201	CATGGGTAATTCGGTTGTTACC	CTAAACATCATGGTGTGACGAAC	331	0.08	
M168	GGTTTGAATGAGACTGGGTCA	TGGTAATCTCATAGGTCTCTGACTG	295	0.08	
M343	AGGTAGGAGGATCCAAAGCTGA	CACCTTTGTCCTCTTGCTCTTT	276	0.08	
M9	TGCAGCATATAAAACTTTCAGGAC	TTCCTCATTTTTGAAGCTCGTG	241	0.08	
M69	TCAGCCATTTCACCAAAACTCT	CTGAAGAAACAAACCTACCTGGAA	233	0.08	
M89	CCAAGCTGGTGAGTCTTATCCT	GCAGAATAGCTGCTCAGGTACA	215	0.08	
M172	CAGAAGATGCCCCATTATATCCT	ACTCCATGTTGGTTTGGAACAG	208	0.08	
M198	TAGGCACTTGGGAACTTACACTC	TTCTTGTGATAGCATGCCGTTT	178	0.08	
SRY10831	CATCCAGTCCTTAGCAACCATT	AATGACACAAGGCACCACATAG	163	0.08	
					
P28	TTTTGAGAGAAGACAAGGGGGATA	TTGGAGGGACATTATTCTCCTGA	559	0.20	Hg-A
M13-M14*	ATCACGCCCCTCTCATTGTC	AGCTCTAGATAAAAGCACATTGACACC	457	0.08	
M91	GATCACAAAGACCTGGACAGATTACA	AACGGAAATGCCAAGAATCGTA	429	0.05	
M31	GCTGAAACAATAGTTCTTCACAATGG	CAGTCCTATGCATAATGCCGTGT	400	0.03	
M114	GCCTTGATTTTCTTCGTACTTCATAAG	CCAGTTTCTCACTGAGTTCATTCCTT	370	0.06	
M28	GGGCTTCAGTTCTTGACGCTAC	CCGTCTTAATTTTGCGGTATTCAA	329	0.04	
M51	AAACCACACCTGTCTTACCAGAGC	CTGTTCCCCAGTTTTCAATCTCC	293	0.04	
M171	GGCTGTGTGGAGTATGTGTTGG	CAAATATCCTGCCCCAGCTTAGT	217	0.04	
M118	TCCCTTGAAATTAAGGACAACAACT	CATTCTTCTCAACCAGCTGACACT	167	0.06	
					
M43	GACTCCATAAGCAAAAGGTCATCAA	AAAAGAAGTTGAGGACTGGAGCA	518	0.12	Hg-B
M112	GGCCATGCTAACAGAGATCTGAC	CACAGTTCAATTCTTGTCTGTTGC	493	0.08	
M152	AAGCAAAAAGCTCCTTCTGAGGT	CAGAAGGTGGATCAGGGTAGAAA	381	0.08	
M182	CATTTTTGTGTCAGGTATCCTTTGT	CAAGACGGCGTATCAACTCAAG	368	0.12	
M108	GCTTTTCTAACACCACCCATGAC	TATGTGATAGAGGTGGCTTTAAGTGG	342	0.08	
M150	CCAGGCTAGCAGTGGAGATGAA	AGGGTGGACTGCTGACCTACTTT	312	0.08	
M146	TTACAGGTGGAATGGGGTGTTAC	GAGAAGAACTGCCTTCCATGACATA	279	0.08	
M60	CCTGATGTGGACTCAACCTTGTA	TGTTCATTATGGTTCAGGAGGAG	250	0.08	
					
M211	CACTGCACACACTACACTGACCAC	ATGTTGATTGGGTAGAGCCCTTT	386	0.06	Hg-B2b
P6	TATTAGGGAAATCACTCAGGATGGT	TCTACGAATGTTTAACCTCAGATACCG	343	0.12	
P8	AGTTGTTGGAAAGCCTCTGTTCA	TGATACTAGACGTGGCATCTTGTCA	313	0.06	
M115	TGCCATGCTTGTTTCTTAATCCA	AACTATGTTGCACATCAGCCTCA	270	0.12	
P7	GGCCAAAGCCTAGAATGAAATTG	AAGTGCTTGTCCAAGGCAGTATAA	228	0.16	
M30	ACAAATCATGAGCTTACAGAACCTG	GGCACAGCCAGATAACCCTACA	200	0.12	
					
M40-M41*	TAGCTGGTATGACAGGGGATGAT	GGGTAGGATAGGCTAGCTATTACGC	435	0.08	Hg-E
M2	GGAGAAGAACGGAAGGAGTTCTAA	ACTTGCCAGAGACTTCCAGTTTG	372	0.12	
M85†	GAACTGGCATCCAATACTAGCTGA	TCACCTCTTTTGTATTGGCTTCTTC	350	0.08	
P2	TGGTCTGGTAACACCCATAAAGGT	GCAGTTTTCTCAGATGCTTCTCCTA	335	0.08	
M35	GCCTAAAGAGCAGTCAGAGTAGAATG	GAGAATGAATAGGCATGGGTTCA	303	0.08	
M75	GTCACATTCCACACATCAAGAAAACT	GTGAATCTCTGCCCAGAAAAGAAAA	274	0.12	
M44	ATTGGATATGGAAGCCAGTCTCA	ATGTGTTTGAGGACCACCCTAGA	250	0.12	
M33	GGCTTCTGTTCAATTTTCCTTTGAT	TTATTTGTTGAAGCCCCCAAGAG	223	0.08	
					
M10	GTTCAAGACAATGAAGGGAGAGACT	TGACATTGACCTGCAGCATAGG	520	0.08	Hg-E1b1ba
M191	GAGCAAGTACAGCGAGCAGTAAG	GGTTTAACACAATGCAGGTCAATTC	480	0.08	
M154†	CAATGGAGGCTATAGGTGATTGC	CTGTTTGTTCATGGAGATGTCTGTA	461	0.08	
M149†	GAACTGGCATCCAATACTAGCTGA	TCACCTCTTTTGTATTGGCTTCTTC	350	0.06	
M116	TATGAAGTACGAAGAAAATCAAGGCTA	TGGGTAGAAAAACTGCAAGTAGATGA	328	0.12	
M58-M155*	TGGGCCTGACCTCTTAACTTGTA	CATAATAAGCTAAGAAACATCCAGCCT	293	0.06	
					
M81†	CAATGGAGGCTATAGGTGATTGC	CTGTTTGTTCATGGAGATGTCTGTA	461	0.10	Hg-E1b1b1
M123-M281*	CTAATTCATGCTCTCAGGGGAAA	ATAACCTCTGGAAGTGTGTCTTTACCT	404	0.10	
M107	AATCCCACCTCACATACACATAAGC	AGGGGTTGACAAGAAAAAGGAATA	386	0.06	
M148†	GAACTGGCATCCAATACTAGCTGA	TCACCTCTTTTGTATTGGCTTCTTC	350	0.08	
M78	ATGGCTGTATGGGTTTCTTTGACT	CGGAATATGGACAGTCATCGTATT	330	0.08	
M165	CAAGTCAGCAAGGAGTAGGTGGA	TTGCACTGACACAAGTTATCTCCCT	293	0.08	
M34	GATAACCTCATTGTGGAGAGCACTT	ATGCTAAAGCAAGTAACCCTGTGG	254	0.10	
M136	ACCAACCGTATTACCTTCTCCTCA	CATGAGTCCAAAGTATAGTGGGCTA	226	0.10	

* Due to the close proximity of the single nucleotide polymorphisms (SNPs), a single amplicon was used.

^† ^Again, a single amplicon was used for these SNPs, but in different assays.

### SBE optimization

The SBE primers designed for the seven SBE assays, ranging from 25 to 80 bases in length, were designed to differ by four to five bases within each assay (see Table 2). This was not always reflected in the electropherogram, with a lack of uniform separation in most of the assays. This resulted in a few extension products (for example, M85 and P2 in Hg-E, M168 and M89 in Hg-YSNP1) co-migrating (see Figure 1). Fortunately, this did not interfere with the interpretation of results. The estimated lengths of extension products in the electropherogram (based on mobility) differed from the designed lengths, on average by four bases. This difference was ascribed to the migration rate of the primer (which was influenced by its actual length), possible secondary structure [11], mobility of the dye attached [12] and the use of POP-7^® ^polymer. This was especially apparent for the M91 primer, a 25-base primer which sized, on average, 11 bases larger. Despite these observations, profiles generated by all the assays were usually easily interpreted.

**Table 2:** Single base extension (SBE) primer sequences and final concentrations used in the study, grouped by assay.

SNP		SBE primer (5' - 3')	Size (mers)	Concentration (μM)	Assay
SRY10831	FW	(C)_3_CTCTTGTATCTGACTTTTTCACACAGT	30	0.10	YSNP1
M168	FW	(C)_12_TGGAGTATGTGTTGGAGGTGAGT	35	0.40	
M89	RV	(GACT)_2_(C)_10_CAACTCAGGCAAAGTGAGAGAT	40	0.40	
M201	FW	(GACT)_2_(C)_9_AGATCTAATAATCCAGTATCAACTGAGG	45	0.40	
M69	FW	(GACT)_4_(C)_11_GGAGGCTGTTTACACTCCTGAAA	50	0.40	
M170	FW	(GACT)_4_(C)_9_ACTATTTTATTTACTTAAAAATCATTGTTC	55	0.80	
M172	FW	(GACT)_7_(C)_12_CCAAACCCATTTTGATGCTT	60	0.40	
M9	FW	(GACT)_8_(C)_11_AAACGGCCTAAGATGGTTGAAT	65	0.40	
M207	FW	(GACT)_8_(C)_11_GCAAATGTAAGTCAAGCAAGAAATTTA	70	0.80	
M198	FW	(GACT)_9_(C)_9_TCAGTATACCAATTAATATTTTTGAAAGAG	75	0.80	
M343	FW	(GACT)_13_(C)_9_AGAGTGCCCTCGTGTTCCA	80	0.40	
					
M91	FW	CCTACATTGCTATTCTGTTTTTTTT	25	0.60	Hg-A
M31	RV	(C)_8_CCACTGCTGTTCTGTCTACCA	29	0.60	
M14	RV	(C)_5_CTTCATTAACTTTTTTAAACTGCTTATA	33	0.60	
M114	RV	(C)_15_AGCTGTACAAGGCTCTTCAAAT	37	0.60	
P28	FW	(C)_14_GGTTAAAAGAAAAAAGCTCTCAGATAG	41	0.40	
M28	RV	(C)_27_TCGAGGTCCTCTGGCATC	45	0.50	
M51	RV	(C)_29_CTCTGATCCCTGTTGGAAGC	49	0.50	
M13	FW	(C)_31_GTAGGTTAAGGGCAAGACGGTTA	54	0.60	
M171	RV	(C)_32_AGGTCTCTGACTGTTCAGTTTTATT	57	0.50	
M118	RV	(C)_35_CAGCTGACACTTGTGTTTTCTTTATA	61	0.20	
					
M60	FW	(C)_3_TTACATTTCAAAATGCATGACTTAAAG	30	0.40	Hg-B
M146	RV	(C)_11_CTAAAACCCAGTGTTAATTACCCG	35	0.80	
M182	FW	(C)_13_CTTAAAGCAGTGGTTAATGTAAACAAA	40	0.80	
M150	FW	(C)_22_TGCCCACACACACAGATAGAAGT	45	0.80	
M152	FW	(C)_23_GCTTTCTCCTGATAATGTTCTTCTTCT	50	0.80	
M108	RV	(C)_27_CTTTTCTCTGACATTCAGGTATAGTTTC	55	0.30	
M43	FW	(C)_39_CTCTTTCCATGGCCAACAAAC	60	0.40	
M112	FW	(C)_38_AAAGAGGTGAGATAAAAACAAAGCAGT	65	0.40	
					
P6	FW	(C)_3_TCAATAGAGGTTTCCACAGTTAAGTCT	30	0.10	Hg-B2b
M115	FW	(C)_3_CAGAGTTTAAATTAGTATTTGATTTCACATTA	35	0.80	
M30	FW	(C)_14_ATCATGTTTTAAGTCCTGACATCTGT	40	0.10	
P7	FW	(C)_21_CCATCACCTGGGTAAAGTGAATTA	45	0.40	
P8	FW	(C)_27_GCAGCTCACCTTTCATTTAGGTC	50	0.20	
M211	FW	(C)_30_TAGGCAAAAGGATGTTAACAACAAG	55	0.80	
					
M40	RV	(C)_10_TCTTCACCCTGTGATCCGCT	30	0.60	Hg-E
M33	FW	CGATCTGTTCAGTTTATCTCATAAGTTACTAGTTA	35	0.30	
M44	RV	(C)_11_AGGAAATCTCCTAACCTTCTAGTACACTG	40	0.40	
M75	FW	(C)_20_AAAAGACAATTATCAAACCACATCC	45	0.10	
M41	FW	(C)_30_TGGCCAACATGGTGAAACTG	50	0.50	
M85	RV	(C)_24_GCTTGTGTTCTATTAAGTGTAGTTTTGTTAG	55	0.20	
P2	RV	(GACT)_8_(C)_8_AGGTGCCCCTAGGAGGAGAA	60	0.40	
M2	RV	(GACT)_9_(C)_6_CCCTTTATCCTCCACAGATCTCA	65	0.60	
M35	RV	(GACT)_10_(C)_9_TTCGGAGTCTCTGCCTGTGTC	70	0.80	
					
M58	FW	(C)_5_ATTTATTGTCTTCTGCAGAATTGGC	30	0.10	Hg-E1b1a
M116	FW	(C)_5_GCTTTCTGAAAAAATAATTTCAAACTGATA	35	0.40	
M149	FW	(C)_9_CTAACAAAACTACACTTAATAGAACACAAGC	40	0.10	
M154	RV	(C)_16_GTGTTACATGGCCTATAATATTCAGTACA	45	0.40	
M155	RV	(C)_23_AATTCAGAATATTTCATCTCTGGTCAC	50	0.40	
M10	FW	(C)_26_AATTTTTTTGTTTATTCCCAATGATCTTA	55	0.50	
M191	FW	(GACT)_5_(C)_10_ATTTACATTTTTTTCTTTACAACTTGACTA	60	0.40	
					
M78	FW	AATTGATACACTTAACAAAGATACTTCTTTC	31	0.80	Hg-E1b1b1
M148	RV	(C)_7_TTTCTAGGTAACGTATGTAGACATTTCTG	36	0.80	
M81	FW	(C)_15_AGAGGTAAATTTTGTCCTTTTTTGAA	41	0.80	
M107	FW	(C)_18_TAAGCCAACGTATTAACCTTCTAATTTC	46	0.20	
M165	RV	(C)_20_AAATATTTCAGGTAAAACCACTCTATTAGTA	51	0.40	
M123	FW	(C)_27_AAAGTCACAGTATCTGAACTAGCATATCA	56	0.80	
M34	FW	(GACT)_7_(C)_13_GCCTGGCTTCCACCCAGGAG	61	0.20	
M136	RV	(GACT)_8_(C)_12_GGTGAGCAGCATTGAGGAAGAC	66	0.10	
M281	RV	(GACT)_8_(C)_11_AGGTTGCACAAACTCAGTATTATTAAAC	71	0.80	

SNP, single nucleotide polymorphism; FW, forward orientation; RV, reverse orientation

While the generation of aspecific peaks did occur occasionally, this was usually due to insufficient purification of the PCR products resulting in the incorporation of the PCR primers or deoxyribonucleotide triphosphates (dNTPs) into the SBE reaction. The presence of one permanent aspecific peak did occur, however, in the Hg-B2b assay (a red peak between the P8 and M211 peaks). This peak seemed to be linked to the P7 primer, perhaps due to a problem during its synthesis, and was usually more visible when overall peak height was decreased.

In order to intensify peak heights, the number of cycles in the SBE reaction program was increased from 25 to 35. While overall peak height improved, variability of peak heights within some assays was unavoidable, despite the adjustment of relative SBE primer concentrations. This was possibly influenced by the efficiency of interaction between SBE primers and template sequences.

### Validation of SBE assays

The seven SBE assays were validated using samples whose haplogroup status was previously determined. A total of 683 samples were then screened. Additionally, sequencing was performed to confirm the presence of alleles for 15 mutations not screened for before the use of these SBE assays. These included M14, M114, M152, P6, P7, P8, M33, M44, M85, M58, M154, M34, M201, M198 and M343.

The marker M91, in the Hg-A assay, is a homopolymer length variant associated with a single base deletion in a poly-T tract [13]. While the use of SBE in the screening of homopolymer variants is not common, the detection of the M91 mutation using the SBE method was successful. This was reaffirmed phylogenetically [14,15] by the presence of this mutation exclusively in samples belonging to subclades of haplogroup A.

The validation process resulted in the redesign of just two SBE primers, P28 and M35. The initial P28 SBE primer did not pick up the mutation, probably due to non-specific primer binding, while the initial M35 primer resulted in an extremely low peak height when the mutation was present. This was possibly due to the preferential amplification of the ancestral allele, or a lower efficiency of binding by the original SBE primer.

Finally, in haplogroups B2b4* and B2b4b, P7 showed the presence of two different extension products, displaying both the ancestral and derived states, simultaneously. This also occurred in haplogroup B2b4a, with P8, additionally, exhibiting the same property. The presence of both states was confirmed when sequencing was performed. Thus, it is likely that all samples in haplogroups B2b4*, B2b4a and B2b4b will display two peaks at the relevant markers. This was, probably, a consequence of these markers being located within paralogous sequence variants [16]. It should be noted that such mutations are more susceptible to back-mutation through gene conversion, as it was with P25 [17]. For this reason, more stable markers that resolve these subclades of B2b4 would be preferable.

### Haplogroup assignment using SBE assays

**Figure 2:**
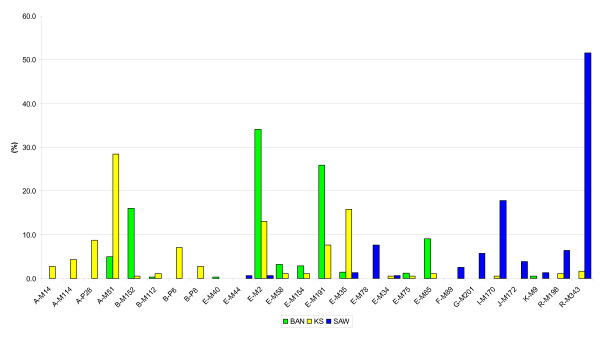
**Clustered column graphs of haplogroup frequencies in south eastern Bantu-speakers (BAN), Khoe-San (KS) and South African Whites (SAW)**.

The sample of 683 males screened using the seven SBE assays was assigned to 26 of the 61 haplogroups that the assays collectively resolved (see Table 3 and Figure 2). The subclades of haplogroup A were found most commonly in the KS, at a frequency of 44.3%. Haplogroup A3b1 was the commonest (28.4%) and was also found to be present in the BAN at low levels (5.0%). The haplogroups A2*, A2a and A2b were found to be unique to the KS at frequencies of 2.7%, 4.4% and 8.7%, respectively. Haplogroup B was present at moderate frequencies in both the KS and the BAN. Its subclades, however, displayed differing distributions, with haplogroup B2a1a occurring at a substantially higher frequency in the BAN (16.0%) than in the KS (0.5%). The situation was reversed with regard to haplogroup B2b, with its subclades together constituting 10.9% of KS individuals, as compared to 0.3% in the BAN. Haplogroup E was the most common haplogroup in the BAN group (78.1%), with its subclades E1b1a* and E1b1a7 occurring at frequencies of 34.1% and 25.9%, respectively. While both these haplogroups occurred in the KS at 13.1% and 7.7%, respectively, the most frequent E subclade amongst the KS was E1b1b1* (15.8%). Haplogroups E2* and E2b1 were found at much higher frequencies (10.2% versus 1.6%) in the BAN compared with the KS. The haplogroups shared between the BAN and KS, described above, showed extremely significant differences in frequency between the two groups (Fisher's exact test: *P *< 0.0001; for haplogroup E2: *P *= 0.0001). Both the KS and the BAN showed low levels (3.3% and 0.6%, respectively) of assimilation of the Eurasian Y chromosome haplogroups I, K* (x R), R1a1, and R1b.

**Table 3:** Y chromosome haplogroup frequencies in south eastern Bantu speakers, Khoe-San, and South African Whites.

Marker	Haplogroup	South eastern Bantu-speakers	(%)	Khoe-San	(%)	South African Whites	(%)
M14	A2* (x A2a, A2b)			5	2.7		
M114	A2a			8	4.4		
P28	A2b			16	8.7		
M51	A3b1	17	5.0	52	28.4		
M152	B2a1a	55	16.0	1	0.5		
M112	B2b* (x B2b1, B2b2, B2b3, B2b4)	1	0.3	2	1.1		
P6	B2b1			13	7.1		
P8	B2b4a			5	2.7		
M40	E* (x E1a, E1b1, E2)	1	0.3				
M44	E1a1					1	0.6
M2	E1b1a* (x E1b1a1, E1b1a2, E1b1a3, E1b1a4, E1b1a5, E1b1a6, E1b1a7)	117	34.1	24	13.1	1	0.6
M58	E1b1a1	11	3.2	2	1.1		
M154	E1b1a4	10	2.9	2	1.1		
M191	E1b1a7	89	25.9	14	7.7		
M35	E1b1b1* (x E1b1b1a, E1b1b1b, E1b1b1c, E1b1b1d)	5	1.5	29	15.8	2	1.3
M78	E1b1b1a					12	7.6
M34	E1b1b1c1* (x E1b1b1c1a)			1	0.5	1	0.6
M75	E2* (x E2a, E2b1)	4	1.2	1	0.5		
M85	E2b1	31	9.0	2	1.1		
M89	F* (x G, H, I, J2, K)†					4	2.5
M201	G					9	5.7
M170	I			1	0.5	28	17.8
M172	J2					6	3.8
M9	K* (x R)†	2	0.6			2	1.3
M198	R1a1			2	1.1	10	6.4
M343	R1b			3	1.6	81	51.6
							
	TOTAL	343		183		157	

^† ^Included in the frequencies of haplogroups F* and K* are those lineages not included in the SBE assays, but typed using PCR or RFLP assays, namely P12F2 and M70.

Y chromosomes in the SAW sample were resolved into macro-haplogroup F (89.2%) of which haplogroup R (58.0%) and haplogroup I (17.8%) together accounted for 75.8%. Haplogroup E comprised the rest of the SAW at 10.8%, with its subclade E1b1b1a found at a frequency of 7.6%. These low to moderate levels of E1b1b1 illustrate the spread of the haplogroup and its subclades into southern Europe and the Middle East, where they are often found. Only two SAW samples, however, clearly belonged to African haplogroups (E1a1 and E1b1a*).

The haplogroup distributions and their relative frequencies in the BAN and the KS were consistent with previous studies which included these populations [18-20], while those of the SAW were found to correlate strongly with the Western European populations from which the majority are derived [21].

## Conclusions

Seven SBE assays containing 60 SNP markers were designed, which allowed for the rapid assignment of samples to Y chromosome haplogroups, more especially those belonging to the major African haplogroups A, B and E. The assays were designed based on markers found in the YCC phylogeny 2003 [10]. Since then many more markers have been discovered that have further resolved the phylogeny [2]. If needed, these new markers could be incorporated into the current SBE assays, thereby increasing resolution. The use of the current SBE assays in screening Y chromosomes, however, has still resulted in increased haplogroup resolution and sample throughput, and at the same time was quicker and made use of less DNA.

Based on the abovementioned haplogroup frequencies, the KS and the BAN populations are discernable from each other with the KS exhibiting significantly higher frequencies of haplogroups A2, A3b1, B2b and E1b1b1. However, the BAN are identifiable by the strong presence of haplogroups B1a1a, E1b1a and E2. The SAW were appreciably different from both the KS and BAN. There were, however, considerable levels of admixture between the populations (especially the KS and BAN) due to their history of interaction. Consequently, while the elucidation of Y chromosome haplogroups is useful in African populations for both anthropological and forensic purposes, their use together with Y chromosome STR screening would considerably improve resolution and thus refine an individual's geographic ancestry.

## Methods

### DNA samples

DNA samples from 683 individuals with diverse ethnic backgrounds were analysed in the present study. All DNA samples were collected with the subjects' informed consent, and this research was approved by the Human Research Ethics Committee (Medical) at the University of the Witwatersrand, Johannesburg (Protocol No. M050906). The sample included 383 south eastern Bantu speakers (BAN), 183 Khoe-San (KS) and 157 South African Whites (SAW).

### DNA extraction

DNA from EDTA-blood was extracted using the salting-out method described by Miller *et al. *[22] and the Gentra Puregene Buccal Cell Kit (Qiagen, Germany) was used to extract DNA from buccal swabs according to the manufacturer's instructions. DNA was quantified using the NanoDrop ND-1000 Spectrophotometer (LabVIEW^®^, Coleman Technologies Inc, FL, USA) and diluted to 10 ng/μL using double distilled water.

### Primer design

The sequences of the regions encompassing the polymorphisms were taken from GenBank. The PCR and SBE primers were designed using Primer3 software [23], before aligning them to human genomic sequences using the National Center for Biotechnology Information basic local alignment search tool (BLAST) in order to confirm template specificity. The screening software, AutoDimer [24] was used to check for primer-dimer and hairpin loop formation. High-performance liquid chromatography-purified primers were purchased via Roche from Metabion (Martinsried, Germany), diluted to 100 μM and frozen.

PCR primer lengths ranged from 20 to 27 mers and GC percentage varied between 30% and 60%. Amplicons were designed to differ slightly in size in order to distinguish them following agarose gel electrophoresis to check the success of the PCR. In total, 53 pairs of PCR primers were designed encompassing all 60 SNPs (see Table 1). Fewer pairs of primers were needed, as some SNPs were co-amplified on the same amplicons (M13 and M14; M40 and M41; M58 and M155; M123 and M281; M81 and M154; M85, M148 and M149).

Poly-C or Poly-GACT tails of differing lengths were added to the 5' end of most SBE primers (Table 2), so as to differentiate between them during capillary electrophoresis. SBE primer lengths ranged from 25 to 80 mers, and differed in size from each other by 4-5 mers.

### Multiplex PCRs

Primer design was verified by performing simplex PCR, using a GeneAmp PCR system 9700 (Applied Biosystems, CA, USA), for each of the primer pairs. Thereafter, the multiplex PCRs were optimized to work with DNA at a concentration of 10 ng/μl (see Table 4), and were catalysed using FastStart Taq DNA Polymerase (Roche, Basel, Switzerland). Relative primer concentrations were adjusted in order to obtain balanced amplification of amplicons within each multiplex. The thermal cycler programmes were as follows: one cycle at 95°C for 6 min, 35 cycles at 95°C for 30 s, 54°C (for YSNP1), 55°C (for Hg-A, Hg-B, Hg-B2b, Hg-E and Hg-E1b1a) or 61°C (for Hg-E1b1b1) for 30 s, extending at 72°C for 30 s and a final extension of 72°C for 10 min. Following the optimization procedures, all multiplex PCRs produced the required amplification products at adequate yields. This was confirmed by running 5 μL of multiplex PCR product on a 2% Metaphore^® ^agarose gel (Cambrex, NJ, USA).

**Table 4:** Polymerase chain reaction (PCR) reagent mixtures for multiplex single base extension assays.

PCR reagent	YSNP1	Hg-A	Hg-B	Hg-B2b
	
	Concentration	Concentration	Concentration	Concentration
DNA	10 ng/μL	10 ng/μL	10 ng/μL	10 ng/μL
Buffer (10×)	1×	0.8×	1×	1×
MgCl_2 _(25 mM)	2 mM	4 mM	3 mM	3 mM
dNTPs 2.5 mM)	300 μM	200 μM	200 μM	200 μM
Forward primer (10 μM)	See Table 1	See Table 1	See Table 1	See Table 1
Reverse primer (10 μM)	See Table 1	See Table 1	See Table 1	See Table 1
FastStart Taq (5 U/μL)	1 U	1 U	1 U	1 U
ddH_2_O	Made up to 25 μL	Made up to 25 μL	Made up to 25 μL	Made up to 25 μL

	**Hg-E**	**Hg-E1b1a**	**Hg-E1b1b1**	
		
**PCR reagent**	**Concentration**	**Concentration**	**Concentration**	

DNA	10 ng/μL	10 ng/μL	10 ng/μL	
Buffer (10×)	1.25×	1.25×	1.25×	
MgCl_2 _(25 mM	3 mM	3 mM	3 mM	
dNTPs (2.5 mM)	200 μM	200 μM	200 μM	
Forward primer (10 μM)	See Table 1	See Table 1	See Table 1	
Reverse primer (10 μM)	See Table 1	See Table 1	See Table 1	
FastStart Taq (5 U/μL)	1 U	1 U	1 U	
ddH_2_O	Made up to 25 μL	Made up to 25 μL	Made up to 25 μL	
				

dNTP, deoxyribonucleotide triphosphate; ddH_2_0, double distilled water

### Multiplex SBE

Excess PCR primers and dNTPs were eliminated from the PCR product mixture, following amplification, using an enzymatic purification method. One unit of Exonuclease I (*Exo I*) and 0.5 units of Shrimp Alkaline Phosphatase (SAP) were added to 5 μL of amplification product and the resultant mixture incubated for 1 h at 37°C, followed by 15 min at 75°C.

The multiplex SBE reactions were performed in a final volume of 5 μL, comprised of 1.5 μL purified amplification product, 1.5 μL of double distilled water, 1 μL of SNaPshot Multiplex Ready Reaction Mix (Applied Biosystems) and 1 μL of SBE primer mix, specific to the assay being conducted (see Table 2 for final primer concentrations). The thermal cycler programme was as follows: 96°C for 10 s, 50°C for 5 s and 60°C for 30 s for 35 cycles.

Following the SBE reaction, excess dideoxyribonucleotide triphosphates (ddNTPs) were removed through the addition of 0.5 U of SAP to the 5 μL SBE product. The mixture was incubated for 1 h at 37°C, followed by 15 min at 75°C.

### Capillary electrophoresis

Following post-extension treatment, 2 μL of SBE product was mixed with 0.5 μL of the internal size standard, GS120LIZ (Applied Biosystems) and 7.5 μL Hi-Di formamide (Applied Biosystems). This was then run on a 3130*xl *Genetic Analyzer (Applied Biosystems). The SNaPshot protocol was originally optimized for use with POP-4 polymer; modifications recommended by Applied Biosystems were incorporated for use of the POP-7 polymer (Applied Biosystems Manual P/N: 4367258). The resultant electropherograms (Figure 1) were analysed using GeneMapperID v3.2 software (Applied Biosystems).

### Assay validations

Some of the markers used in the SBE assays were validated using a set of control samples, previously screened using RFLP assays. Those markers for which samples of known haplogroup were unavailable were sequenced in order to confirm the presence of the polymorphism. After the screening of the 683 samples, Fisher's exact tests were performed using GraphPad InStat version 3.10 32 bit for Windows (GraphPad Software, CA, USA, http://www.graphpad.com), in order to test significance of differences in haplogroup frequency between the BAN and KS.

## Abbreviations

BAN: Bantu speaker; dNTP: deoxyribonucleotide triphosphate; ddNTP: dideoxyribonucleotide triphosphate; ddH20: double distilled water; KS: Khoe-San; PCR: polymerase chain reaction; RFLP: restriction fragment length polymorphism; SAP: shrimp alkaline phosphatase; SAW: South African White; SBE: single base extension; SNP: single nucleotide polymorphism; STR: short tandem repeat.

## Competing interests

The authors declare that they have no competing interests.

## Authors' contributions

TN conceived the study, participated in its design, carried out screening of samples and drafted the manuscript. CMS and HM were involved in sample collection, screening, and assisted with the manuscript. PP, RM and JCE were involved in sample collection and screening. HS participated in its design and contributed to the manuscript. All authors read and approved the final manuscript.
